# Immunotherapy in the context of immune-specialized environment of brain metastases

**DOI:** 10.1093/discim/kyad023

**Published:** 2023-11-16

**Authors:** Fiona James, Mihaela Lorger

**Affiliations:** School of Medicine, University of Leeds, Leeds, UK; School of Medicine, University of Leeds, Leeds, UK

**Keywords:** brain metastases, immunotherapy, brain immune privilege

## Abstract

Brain metastases (BrM) develop in 20–40% of patients with advanced cancer. They mainly originate from lung cancer, melanoma, breast cancer, and renal cell carcinoma, and are associated with a poor prognosis. While patients with BrM traditionally lack effective treatment options, immunotherapy is increasingly gaining in importance in this group of patients, with clinical trials in the past decade demonstrating the efficacy and safety of immune checkpoint blockade in BrM originating from specific tumor types, foremost melanoma. The brain is an immune-specialized environment with several unique molecular, cellular, and anatomical features that affect immune responses, including those against tumors. In this review we discuss the potential role that some of these unique characteristics may play in the efficacy of immunotherapy, mainly focusing on the lymphatic drainage in the brain and the role of systemic anti-tumor immunity that develops due to the presence of concurrent extracranial disease in addition to BrM.

## Introduction

Brain metastases (BrM) have the highest frequency among intracranial tumors. They originate from different primary tumor types, with the highest incidence being observed in lung cancer, melanoma, breast cancer, and renal cell carcinoma [1]. Patients with BrM have a poor prognosis, although overall survival strongly depends on cancer type and molecular profile. Treatment options for BrM are limited and include local therapies such as surgery, radiotherapy, and radiosurgery, as well as systemic therapies, including immunotherapy [2, 3]. The latter will be the focus of this review. While the brain has been previously thought to be immune-privileged, it is now well accepted as an immune-specialized environment with several immunologically distinct compartments. This includes the ventricles, where efficient immune responses against foreign grafts (i.e. normal or cancerous foreign tissue) are initiated, and the brain parenchyma, where foreign grafts escape the immune surveillance [4–6]. After providing a brief overview of the status of immunotherapy for BrM in the clinic, we will focus on the concepts and features that are unique to BrM and were revealed mainly through preclinical studies. We will focus on tumors within the immune-specialized brain parenchyma.

## Immunotherapy for brain metastases in the clinic

Immuno-oncology (IO) has revolutionized the treatment of many different cancer types, bringing the potential for long-term, durable responses. IO drug development has become a large and fast-growing field with a 91% increase in number of drugs in development between 2017 and 2019 [7]. These drugs are now licensed as treatments for many different tumor types, and this is increasing every year. Along with different tumor types their scope is steadily increasing from the original trials in late-stage, unresectable melanoma, through to first-line treatment in metastatic disease and even into the adjuvant/neo-adjuvant setting. Indications of FDA-approved ICI with the level of evidence were recently reviewed by Vaddepally *et al*. [8].

Historically, the use of IO was withheld from BrM patients through fears of severe neurological complications, due to the immune-specialized environment of the brain and many original trials excluded this critical population entirely. Recently, with growing preclinical evidence, these patients have been increasingly recognized as having huge potential to benefit from this revolution. Recently efforts have been made to improve trial enrollment of this underserviced population with many trials seeking to include patients with BrM if these are asymptomatic [9, 10]. Issues remain about the paucity of BrM-exclusive clinical trials and inclusion of BrM-specific outcome measures in larger trials. BrM management is also complicated by consideration of concomitant extracranial disease control.

## Immune checkpoint inhibition

Immune checkpoints are immune inhibitory receptors expressed on immune cells, most notably T cells, that restrain the immune system. Clinically most relevant immune checkpoints are programmed cell death protein 1 (PD-1) and cytotoxic T lymphocyte-associated protein 4 (CTLA-4). Other T-cell immune checkpoints that are being actively explored as therapeutic targets include lymphocyte-activation gene 3 (LAG-3) and T-cell immunoglobulin and mucin domain-containing protein 3 (TIM-3), as well as immune checkpoints expressed on natural killer (NK) cells, such as NKG2A and TIGIT. Characteristics and mechanisms of action of individual immune checkpoints have been extensively reviewed elsewhere [11]. Blocking these receptors with targeted monoclonal antibodies can unleash the body’s immune system and lead to long-term tumor control. However, a high proportion of patients fail to respond [12], limiting the impact of these life-changing therapies.

In intracranial disease, early trials investigating single-agent immune checkpoint inhibition (ICI) with ipilimumab (anti-CTLA-4) in melanoma BrM showed some efficacy with 18–24% intracranial response rate [13, 14]. PD-1 blockade with pembrolizumab or nivolumab showed a slightly higher response rate in BrM in melanoma patients [15, 16]. Of note, a better response rate is demonstrated in those patients who are asymptomatic from their intracranial disease, particularly when a more specific revised definition of symptomatic central nervous system (CNS) metastases is retrospectively applied [17]. Following successful trials at extracranial sites, increased efficacy has been demonstrated in the brain from combined ICI with PD-1/CTLA-4 blockade with response rates approaching 50% in these selective cohorts of patients with melanoma BrM, with some patients achieving a complete response [18, 19]. There is increasing evidence for the benefit of combining these therapies with radiotherapy, with a recent meta-analysis of 44 studies showing improvement in survival outcomes and acceptable safety profile in melanoma BrM [20].

Several trials have been done in metastatic non-small cell lung cancer with a recent meta-analysis of 10 phase-three trials showing that regimens containing ICI improved progression-free survival (HR 0.53, 95% CI 0.40–0.69, *P* < 0.01) versus chemotherapy alone [21].

In breast cancer, despite an absence of BrM-specific clinical trials, the inclusion of small numbers of patients with asymptomatic, previously treated BrM into the larger landmark trials of immune checkpoint blockade in advanced disease have suggested safety despite inclusion of patients with BrM [22, 23].

## Cellular immunotherapies for BrM

In adoptive cell therapies, the immune cells isolated from patients or healthy donors are expanded ex vivo and infused back into cancer patients. Adoptive cell therapies are focusing mainly on T cells, NK cells, and dendritic cells (DCs). These cells can be also genetically modified prior to their reinfusion. The most prominent example is the expression of chimeric antigen receptors (CARs) in T cells. CARs recognize a defined antigen expressed on cancer cells, directing the cytotoxic activity of T cells toward the latter. CAR T cells have shown huge promise in hematological malignancies and are currently being trialed in some solid tumor types [24]. No late-phase studies have been done on patients with BrM, however, a phase I trial study is currently recruiting to investigate the side effects and best dose of HER2-CAR T cells injected intra-ventricularly in treating patients with brain or leptomeningeal metastases from HER2+ breast cancer (https://clinicaltrials.gov/ct2/show/NCT03696030).

Another example of cellular immunotherapies are DC vaccines. Cancer vaccines employ the principle that injecting cancer antigens or cells alongside an immune adjunct can boost the immune system to recognize and respond to cancer. There is currently a completed trial with results awaited from PERCELLVAC3 study, where patients with BrM receive vaccines consisting of mRNA tumor antigen-pulsed DCs [25]. Also in recruitment is a phase IIa study of DC vaccines against HER2/HER3 and pembrolizumab in patients with asymptomatic brain metastasis from triple negative breast cancer or HER2+ breast cancer (https://classic.clinicaltrials.gov/ct2/show/NCT04348747). Substantial evidence for the safety of CAR T-cell therapies and DC vaccines in the CNS also comes from glioma [26, 27].

## Insights from preclinical studies

### Central nervous system immunology

**Figure 1:**
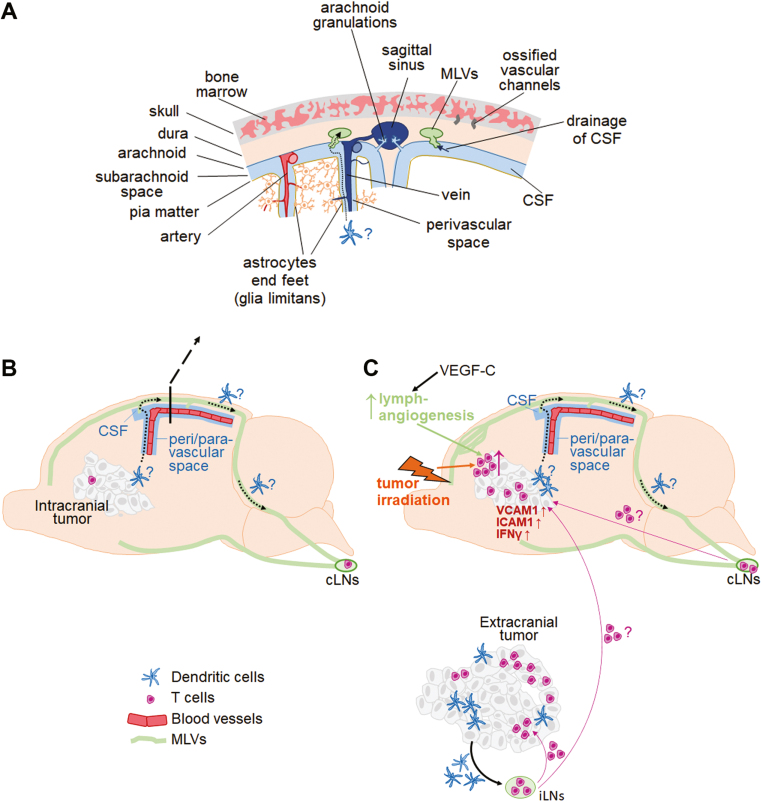
the role of different central nervous system (CNS) compartments, the lymphatic system and systemic immunity in immunobiology and response to immunotherapy in brain metastases. (A) CNS compartments relevant to the tumor immunology in the brain are illustrated. Arrows indicate drainage of cerebrospinal fluid (CSF) from the subarachnoid space into the meningeal lymphatic vessels (MLVs; solid line) and a potential route for dendritic cell (DC) migration from within the brain parenchyma to the subarachnoid space and further into MLVs (dotted line). (B and C) Tumor microenvironment in the brain in the context of immune checkpoint blockade is depicted in the absence (B) and presence (C) of extracranial tumor, illustrating different abundance in T cells and dendritic cells (DCs) within intracranial tumors in the presence versus absence of extracranial disease. Possible recruitment of T cells to BrM from extracranial-TDLNs and cLNs is indicated. Association between the perivascular spaces within brain parenchyma and meningeal lymphatic vessels is shown. A potential route of DC migration from BrM to the cLNs is indicated with dotted arrows. Irradiation and VEGF-C-enhanced lymphangiogenesis are highlighted as examples of stimuli known to enhance T-cell abundance in brain metastases in (C).

One of the initial pieces of evidence suggesting that the brain is immunologically distinct was provided over 100 years ago by Shirai *et al*. [28], who observed that mouse sarcoma tumors grew successfully in rat brains, while being recognized as a foreign tissue and were rejected when growing under the skin. This led to an assumption that the brain is immune-privileged and separated from the immune system. New discoveries over the past decades, however, revealed several distinct compartments involved in the interactions between the CNS and the immune system. These compartments harbor a variety of immune cell types and are located primarily along the CNS borders, including the meninges, choroid plexus, and perivascular spaces (reviewed in [29]). Briefly, the choroid plexus is located within the ventricles, it consists of specialized cells and one of its main functions is to produce cerebrospinal fluid (CSF). Perivascular spaces are CSF-filled compartments surrounding small blood vessels within the brain. Lastly, meninges are membranous structures that surround the brain and consist of three layers: the pia matter adjacent to the brain parenchyma, the middle layer called arachnoid matter, and dura matter which is the closest to the skull. Arachnoid and pia matter enclose the subarachnoid space, which is filled with CSF. Dura contains venous sinuses, which serve as regional immune cell hubs [30]. Dural sinuses lay in parallel to the recently rediscovered meningeal lymphatic vessels (MLVs) (see Fig. 1A for a detailed illustration of some of these compartments).

Despite occasional literature reports of lymphatic vessels at the dura [31], until recently there was a dogma in the field that CNS lacks lymphatic vessels. However, in 2015, two important studies unequivocally demonstrated the existence of MLVs in mice [32, 33], followed by a study demonstrating the presence of MLVs in humans [34]. The rediscovery of MLVs was very important because lymphatic vessels play an essential role in mounting of immune responses against antigens, including tumor antigens. Cancer-immunity cycle consists of several steps that need to be executed before anti-cancer immune response is initiated (reviewed in [35, 36]). Briefly, tumor antigens are released within tumors and captured by DCs, which are professional antigen-presenting cells. DCs then migrate along lymphatic vessels to the regional tumor-draining lymph nodes (TDLNs), where they present the captured antigens on MHCI and MHCII molecules to T cells, resulting in priming and activation of T cells. Subsequently, the activated T cells are home to tumors, where they can potentially recognize and eliminate cancer cells. Recent evidence from extracranial tumors suggests that following their initial activation in TDLNs, CD8+ T-cell differentiation into effectors occurs within tumors in a second step that also requires an involvement of DCs [37].

MLVs are present at the dorsal and basal part of the skull and have been shown to contribute to antigen drainage from the CSF to the cervical lymph nodes (cLNs) (Fig. 1A, B). In contrast, brain parenchyma lacks classical lymphatic vessels. Instead, distinct subsets of spaces along the brain blood vessels have been proposed to serve as pseudolymphatics, used for removal of metabolic waste products through efflux of interstitial fluid from the brain parenchyma to the CSF, as well as for the delivery of fresh CSF to the brain interstitium. The exact identity of these spaces is still a matter of debate (reviewed in [38]), with the glymphatic system model considered to be most likely at present. This model proposes that CSF enters brain parenchyma through the arterial perivascular spaces, and interstitial fluid leaves the parenchyma through the venous perivascular spaces [39] (Fig. 1A). The latter are most likely also the route of drainage for soluble antigens, which then enter MLVs together with the CSF, while it remains unclear whether the same route is used for the migration of DCs to the MLVs and further to the cLNs (Fig. 1A and B).

The skull bone marrow (BM) represents a further, recently discovered, immunological compartment that functions as an interface between the CNS and the immune system [40]. Under homeostasis, different types of myeloid cells in the meninges, including the Ly6C^+^ monocytes, neutrophils, and DCs, are mostly derived locally from the skull BM rather than from the blood, and can migrate from the skull BM directly into meninges via ossified vascular channels [40, 41] (Fig. 1A). The latter also permit influx of CSF into the skull BM, providing means for CNS antigens contained in the CSF to imprint the BM immune cells. Interestingly, a less pro-inflammatory molecular profile was observed in the skull-derived as compared to the blood-derived myeloid cells in the context of experimental autoimmune encephalomyelitis, suggesting that molecular characteristics of myeloid cells may contribute to the immune-specialized properties of the brain [40]. However, the contribution and characteristics of the skull BM-derived myeloid cells in brain metastases remain to be elucidated.

In summary, while the existence of diverse interactions between the CNS and the immune system is now well accepted, it remains that the immune responses in the brain are regulated differently than outside the CNS. This immune-specialized status is thought to be due to a combination of factors, including characteristics of the cellular microenvironment, such as myeloid cells, and characteristics of the lymphatic system and the CNS-draining lymph nodes, which will be further discussed in the context of brain metastases below. Of note, in contrast to primary brain cancer and non-cancerous CNS disorders, BrM develops in the presence of immune responses against extracranial disease. This co-existence of immune responses directed against tumors within an immune-specialized brain microenvironment and “immune-competent” extracranial environment represents a unique situation that requires additional considerations.

## The role of extracranial disease and systemic immunity in immunotherapy in BrM

As mentioned earlier, in contrast to the foreign grafts implanted under the skin, the foreign grafts implanted into the brain escape immune surveillance. However, when foreign tissue implanted under the skin was spontaneously rejected, this led to the rejection of this same tissue implanted into the brain of the same animal [5, 42], suggesting that the immunity spreads from the extracranial to the intracranial location. Unlike foreign tissue, intracranial and extracranial tumors in metastatic melanoma are immunologically compatible with their host and tumors evade anti-tumor immune responses as one of the hallmarks of cancer via a variety of mechanisms (reviewed in [43]). Growth of melanoma BrM in experimental models seems to be unaffected by the presence of subcutaneous tumors of the same origin [44, 45]. However, this changes in the context of immunotherapy, where the growth of BrM is impacted by the extracranial tumor. Our preclinical study demonstrated that combined PD-1/CTLA-4 blockade fails to reduce intracranial melanoma growth when mice are bearing tumors only in the brain. In contrast, a significant reduction in intracranial melanoma growth upon treatment was observed when mice were bearing subcutaneous tumors in addition to tumors in the brain [45], with response rates in line with the clinically observed intracranial efficacy of this combination therapy in melanoma patients [18, 19]. The requirement of extracranial tumor for the efficacy of ICI in melanoma BrM was also observed in a subsequent preclinical study by Song *et al*. [46]. Thus, the efficacy of combined anti-PD-1/anti-CTLA-4 therapy in BrM seems to rely upon the presence of the extracranial disease, and therefore it is important to mimic this in pre-clinical models for the purpose of studying the disease and therapies. There is a scope to further modify this model, by resecting the extracranial tumor prior to inducing BrM, to mimic primary tumor removal performed in melanoma and breast cancer patients in the clinic. However, patients with BrM commonly harbor extracranial metastases even after the primary tumor has been removed, and therefore a model without extracranial tumor resection may better mimic the clinical situation.

Mechanistically, extracranial tumor is required for the therapy-induced systemic increase in CD44+CD62L− effector CD8+ T cells and therapy-induced infiltration of CD8+ T cells into intracranial melanoma tumors, accompanied by increase in IFNγ expression and upregulation of vascular T-cell entry receptors ICAM1 and VCAM1 in tumors [45] (Fig. 1C). These observations suggest that systemic immunotherapy generates an immune response directed against extracranial lesions, which subsequently spreads to the intracranial tumor. Further evidence for this comes from studies demonstrating that local induction of immune responses against extracranial tumors leads to immune responses directed against tumors in the brain. For example, local therapy of orthotopic AT-3 triple-negative breast tumors with radiation, Flt3 and TLR3/CD40 stimulation resulted in a reduced BrM burden following a systemic cancer cell seeding, and this local therapy synergized with systemic PD-L1 blockade [47].

Patients that initially present early with primary tumors such as melanoma and breast cancer will mostly undergo surgical resection of their tumors, but remain at risk of developing metastases, including in the brain. The question in this context is whether a potential immune memory response induced by immunotherapy before clinically detectable metastases develop is sufficient to drive anti-tumor immune responses against BrM that occur later. There is some experimental evidence suggesting this may be the case. When subcutaneous B78 melanoma tumors were rejected following treatment with a combination of radiation, anti-CTLA-4 and intra-tumoral injection of an immunocytokine (IL-2 fused to an antibody targeting disialoganglioside GD2), this led to rejection of intracranial tumors implanted 120 days post-rejection of extracranial tumors and was accompanied by enhanced infiltration of CD4+ and CD8+ T cells [48]. Another study has shown that a rejection of subcutaneous melanoma tumors following intra-tumoral injection of IFNβ-expressing insect cells inhibits subsequent colonization of the brain by the same melanoma cell line, and depletion of CD4+ and CD8+ T cells abrogated this effect [44]. Furthermore, a rejection of orthotopic EMT6 breast tumors following a local CpG therapy prevented intracranial growth of the same cell line [49]. These observations suggest that treatment-induced T-cell-dependent immune memory against extracranial tumors may have a potential to reduce metastatic recurrence in the brain. Notably, adjuvant pembrolizumab therapy in patients with complete resection of cutaneous melanoma metastatic to lymph node increased distant metastases-free survival at a 3.5-year median follow-up from 49.4% to 65.3% [50]. Furthermore, adjuvant pembrolizumab in this study reduced the proportion of patients with recurrence specifically in the brain from 7% to 5%, providing some evidence for a potential benefit of adjuvant immunotherapy in patients at risk of developing BrM.

Overall, experimental evidence demonstrates that while immune responses against intracranial tumors are limited when the only tumor site is intracranial, an effective immune attack on BrM can be unleashed through the development of systemic immunity against extracranial tumors with shared tumor antigens.

## The role of meningeal lymphatic vessels in immune responses and immunotherapy in BrM

The rediscovery of MLVs prompted studies into their role in immune responses to tumors in the brain. It has been observed that intracranial melanoma growth enhances lymphangiogenesis of dorsal MLVs in mice simultaneously bearing intracranial and subcutaneous tumors, while only a mild remodeling of basal MLVs occurred [51]. Chemical ablation of dorsal MLVs in this model significantly reduced the size of deep cLNs and to a lesser extent superficial cLNs. It further reduced drainage of intra-tumoraly injected dextran and the abundance of antigen-presenting (CD11c+MHCII+) DCs that have taken up intra-tumoraly injected FITC-labeled beads to the deep cLNs. This indicates that MLVs are required for efficient drainage and DC migration from intracranial tumors to the cLNs. However, the exact route of DC migration, and whether this occurs via MLVs, remains to be elucidated (Fig. 1A and, B). In line with the above, ablation of MLVs reduced the efficacy of combined PD-1/CTLA-4 blockade in mice simultaneously bearing intracranial and extracranial melanoma tumors [51].

As an alternative approach to studying the role of MLVs, Song *et al*. [46] administered a vector expressing VEGF-C mRNA into cisterna magna to experimentally potentiate lymphangiogenesis in the meninges. This study focused primarily on glioma, showing that VEGF-C-induced angiogenesis enhances T-cell abundance in tumors and efficacy of ICI, and these findings could be reproduced in a brain metastases model. Interestingly, in the context of ICI, VEGF-C-induced expansion of MLVs was able to compensate for the lack of the extracranial disease in a melanoma BrM model, resulting in intracranial efficacy of combined PD-1/CTLA-4 blockade in the absence of extracranial tumors [46], while no further enhancement of intracranial ICI efficacy through VEGF-C was observed in mice bearing subcutaneous tumors in addition to intracranial tumors. Further analysis suggested that VEGF-C-mediated expansion of MLVs enhances T-cell priming in the cLNs. Thus, MLVs seem to mediate increase in intra-tumoral T cells as well as an increase in DC abundance and T-cell priming in TDLNs. Further studies into MLVs and DC migration in the context of BrM, especially their route from tumors within the brain parenchyma to the CSF in subarachnoid space, from where they could enter MLVs (Fig. 1A), are required to fully understand to what extent the specifics of the lymphatic drainage system in the brain contribute to the immune-specialized status of this organ.

In summary, the preclinical data suggest that intracranial efficacy of ICI relies on the enhancement of T-cell priming/infiltration, or enhancement in tumor antigen drainage. This can be caused by the presence of the extracranial disease, which is a natural occurrence in most melanoma patients, or be induced through stimuli such as VEGF-C [46] or irradiation (reviewed in [52]) (Fig. 1C).

## Characteristics of cervical lymph nodes

Like cancer-unrelated CNS antigens, antigens originating from brain tumors are also predominantly found in the cLNs. T-cell proliferation following intracranial tumor growth is also induced mainly in cLNs [6, 53, 54], demonstrating that cLNs are the TDLNs for intracranial tumors. Experimental evidence suggests that not all LNs have the same characteristics, and this is likely to influence anti-tumor immune responses. For example, LNs at different anatomical locations have been shown to support the upregulation of distinct T-cell surface receptors in a site-specific manner. Intracranial growth of M57 fibrosarcoma, which induces a spontaneous tumor rejection, induced a different pattern of T-cell surface receptor expression in cLNs as compared to the pattern induced by subcutaneous and intraperitoneal tumor growth in inguinal and mesenteric LNs. Furthermore, T cells that were primed within cLNs homed more efficiently to tumors in the brain than T cells that were primed in inguinal LNs. The main receptors upregulated on T cells in cLNs were VLA-4, P- and E-selectin, and T-cell homing to intracranial tumors required the VLA4 subunit integrin α4. Thus, T cells primed in TDLNs at different locations seem to be equipped with distinct site-specific homing phenotypes [54].

There is also evidence that cLNs support the development of tumor tolerance. When cancer cells were injected under the skin, into the brain ventricles or into the brain parenchyma, the latter resulted in the strongest accumulation of tumor-derived antigens in parotid and deep cLNs. Interestingly, this correlated with increased numbers of myeloid-derived suppressor cells and decreased numbers of CD8+ T cells in intracranial tumors, suggesting the development of immunotolerance instead of an effective anti-tumor immune response [6]. In line with this, it has been shown that cLNs dictate the development of delayed-type hypersensitivity to injected peptides, leading to tolerance towards peptides that are being delivered via the nasal route [55]. Consequently, it has been suggested that cLNs may be more prone to induce tumor tolerance as compared to the LNs at other locations [6, 55, 56]. In this context, the requirement of extracranial tumor for the intracranial ICI efficacy may be explained by the ability of effective anti-tumor immune responses that are generated at extracranial locations, to overcome tumor tolerance induced in the cLNs.

## Conclusions

In patients with BrM, brain tumor is located within the immune-specialized environment, while, in general, simultaneously cancer lesions with shared tumor antigens are present at “immune-competent” extracranial locations. These circumstances differ to the primary brain cancer such as glioma, where tumors are present exclusively in the immune-specialized intracranial environment, as well as to the extracranial tumors (primary and metastatic) residing solely in “immune-competent” environments, and thus represent a unique situation found only in BrM. This review mainly discusses how these BrM-specific circumstances lead to unique interactions between anti-tumor responses generated against the extracranial disease and BrM, and how this is impacted by the characteristics of the brain-associated lymphatic system. There are other important factors that need to be considered in the context of the therapeutic response to immunotherapies in the brain that have not been discussed in this review, such as lower abundance of DCs and CD8+ T cells in BrM as compared to extracranial tumors [57, 58], differences in molecular profiles between BrM and extracranial disease [59], which may impact on anti-tumor immune responses, the presence of the blood-tumor barrier that originates from the specialized blood-brain barrier [60], and brain-resident stroma including microglia and astrocytes [2]. Currently explored immunotherapies could overcome some of these factors. For example, a low abundance of CD8+ T cells in BrM could be potentially overcome through intrathecally administered adoptive T cell or CAR-T-cell therapy. CAR-T-cell therapy against BrM-specific antigens could be further employed to overcome limitations potentially imposed by differences in molecular profiles between BrM and extracranial tumors, considering that extracranial tumors may be more “visible” to the immune system than tumors in the brain, and consequently the systemic anti-tumor responses may “overlook” key BrM-specific tumor antigens. DC vaccines could enhance T-cell priming in cervical LNs, which may be an important step as suggested by the data from preclinical studies discussed in this review. While one could speculate that the lack of classical lymphatic drainage in the brain parenchyma limits DC migration from tumors in the brain to the cLNs, our understanding of this process is currently incomplete, and a better understanding is needed before appropriate strategies addressing this can be explored. Overall, understanding how features that are unique to BrM affect the efficacy of immunotherapies in the brain is key to revealing opportunities for interventions that can enhance therapeutic outcomes in this much needed area.

## Data Availability

N/A

## Abbreviations

BrM: brain metastases
CARs: Chimeric antigen receptors
cLNs: Cervical lymph nodes
CNS: central nervous system
CSF: Cerebrospinal fluid
CTLA-4: cytotoxic T lymphocyte associated protein 4
DC: Dendritic cell
FDA: food and drug administration
HER2: Human epidermal growth factor receptor 2
ICI: immune checkpoint inhibition
IO: immuno-oncology
LAG-3: lymphocyte-activation gene 3
MLVs: Meningeal lymphatic vessels
NK: natural killer
PD-1: programmed cell death protein 1
PD-L1: Programmed cell death protein ligand 1
TDLNs: Tumor draining lymph nodes
TIM-3: T-cell immunoglobulin and mucin domain containing protein 3.

## Permission to Reproduce

N/A

## Clinical Trial Registration

N/A
